# Role and mechanism of KIAA1429 in regulating cellular ferroptosis and radioresistance in colorectal cancer

**DOI:** 10.17305/bb.2024.10313

**Published:** 2024-12-01

**Authors:** Hao Chen, Peipei Zhu, Dan Zhu, Juan Jin, Qianni Yang, Xiaodong Han

**Affiliations:** 1 Department of Gastroenterology, Dazhou Hospital of Integrated Traditional Chinese and Western Medicine, Dazhou, China; 2 Department of Oncology, Linyi Third People’s Hospital, Linyi, China; 3 Department of Gastroenterology, Shanxi Cancer Hospital, Taiyuan, China; 4 Gynecological Radiotherapy Ward, Shanxi Cancer Hospital, Taiyuan, China

**Keywords:** Colorectal cancer, ferroptosis, radioresistance, KIAA1429, lncRNA EBLN3P, miR-153-3p, γ-H2AX, m6A

## Abstract

Colorectal cancer (CRC) is one of the most common non-cutaneous malignancies, causing significant mortality and a substantial burden. This study aims to explore the role of KIAA1429 (also known as vir-like m6A methyltransferase-associated [VIRMA]) protein in the radioresistance of CRC. CRC cells and a radioresistant cell line were cultured, and KIAA1429 expression was detected. After the downregulation of KIAA1429, its effect on the radioresistance and ferroptosis of cancer cells was analyzed. The role of ferroptosis in radioresistance was verified. The binding relationship among long non-coding RNA endogenous Bornavirus-like nucleoprotein 3, pseudogene (lncRNA EBLN3P), microRNA (miR)-153-3p, and KIAA1429 was analyzed. KIAA1429 and lncRNA EBLN3P were highly expressed in CRC, while miR-153-3p was poorly expressed. KIAA1429 and lncRNA EBLN3P were further increased/decreased in the radioresistant cells. KIAA1429 knockdown decreased the survival rate of the radioresistant cell line after X-ray irradiation and increased gamma H2A histone family member X (γ-H2AX), ferroptosis, and oxidative stress. A ferroptosis inhibitor alleviated the inhibitory effect of KIAA1429 knockdown on radioresistance. KIAA1429-mediated m6A modification up-regulated lncRNA EBLN3P, and lncRNA EBLN3P increased KIAA1429 by competitively binding to miR-153-3p. miR-153-3p silencing or lncRNA EBLN3P overexpression attenuated the promotion of ferroptosis and the inhibition of radioresistance induced by KIAA1429 knockdown. Overall, KIAA1429-mediated m6A modification upregulated lncRNA EBLN3P expression, and lncRNA EBLN3P increased KIAA1429 expression by competitively binding to miR-153-3p, thus reducing ferroptosis and increasing the radioresistance of CRC.

## Introduction

Colorectal cancer (CRC) ranks as the third most commonly diagnosed cancer and the second most common cause of cancer-associated death worldwide [1]. In 2023, approximately 153,020 individuals were diagnosed with CRC, and 52,550 died from the disease, including 19,550 cases and 3750 deaths among individuals younger than 50 years [2]. It is estimated that the number of new CRC cases will increase by 63%, reaching 3.2 million per year by 2040, while the mortality rate is projected to rise by 73%, to 1.6 million per year [3]. Various genes and the interaction of multiple pathways have been implicated in the oncogenesis of CRC, although the complex mechanisms remain incompletely understood [4]. The development of CRC is a multistep process initiated by benign polyps, which may progress to cancer through interactions between environmental and genetic factors [5]. Additionally, abnormal cell proliferation, cell differentiation, resistance to apoptosis, invasion of adjacent structures, and distant metastasis are associated with CRC carcinogenesis, mechanisms of which are complex and not yet fully elucidated [3]. Primary treatment methods for CRC include surgery, chemotherapy, radiotherapy, immunotherapy, and targeted therapy; however, they all have their shortcomings [6]. Consequently, CRC presents a global public health challenge in terms of morbidity, mortality, and the availability of healthcare services [7].

N6-methyladenosine (m6A) is the most common, abundant, and conserved internal transcriptional modification, and m6A modification is installed by m6A methyltransferases [8]. KIAA1429 (vir-like m6A methyltransferase associated) is a major m6A methyltransferase that plays significant biological and pharmacological roles in human diseases [9]. KIAA1429 is associated with various biological behaviors, including pathways related to benign/poorly differentiated tumors and tumor metastasis [10]. In particular, KIAA1429 has been found to regulate aerobic glycolysis in CRC in a hexokinase 2 (HK2)-dependent manner [11]. In addition to glycolysis, KIAA1429 can modulate ferroptosis in oral squamous cell carcinoma cells, and ferroptosis may represent a promising target in tumor resistance to therapy, including radiotherapy [12, 13]. However, the role of KIAA1429 in the radiotherapy resistance of CRC remains unknown.

Long non-coding RNAs (lncRNAs) are RNA transcripts longer than 200 nucleotides (nt) that do not encode proteins and are functional units themselves [14]. Previous research has demonstrated that lncRNAs play a crucial role in CRC [15]. For instance, lncRNA endogenous bornavirus-like nucleoprotein (EBLN3P) stimulates CRC progression by regulating U2AF homology motif kinase 1 (UHMK1) expression via sponging miR-323a-3p [16]. This reflects the involvement of lncRNAs in CRC progression through coordination with microRNAs (miRNAs), a group of small non-coding RNAs that post-transcriptionally control gene expression by targeting mRNAs [17, 18]. Additionally, lncRNA ZNFX1 antisense RNA 1 (ZFAS1)-mediated miR-153-3p has been implicated in CRC cell growth and metastasis [19]. Nonetheless, the interaction between lncRNA EBLN3P and miR-153-3p has not yet been investigated. Given that m6A regulators can influence CRC development by regulating lncRNAs and that lncRNAs can modulate m6A modification by interacting with m6A regulators [20], this study aims to explore the functions of KIAA1429, lncRNA EBLN3P, and miR-153-3p in CRC from the perspective of radioresistance, thereby providing potential therapeutic targets for CRC patients.

## Materials and methods

### Cell culture

The normal intestinal epithelial cell line (NCM460) and CRC cell lines (HCT116, SW620, LoVo) were procured from the American Type Culture Collection (ATCC) and cultured in Dulbecco’s modified Eagle medium (DMEM) containing 10% fetal bovine serum (FBS) and 1% antibiotics at 37 ^∘^C with 5% CO_2_ and 95% air.

### Establishment of radioresistant cell lines

Radioresistant cells were established according to a previously described method [21]. When HCT116 and SW620 cells reached 50% confluence, they were irradiated at 4 Gy. The cells were then repeatedly irradiated with 4 Gy until the total radiation dose reached 40 Gy. The resulting radioresistant cell lines were named HCT116R and SW620R.

### Cell transfection

The EBLN3P overexpression vector (EBLN3P) and empty vector (NC) were obtained from Shanghai GenePharma (Shanghai, China). KIAA1429 small interfering RNAs (siRNAs) (si-KIAA1429-1, si-KIAA1429-2, and si-KIAA1429-3), EBLN3P siRNAs (si-EBLN3P-1 and si-EBLN3P-2), negative control siRNA (si-NC), miR-153-3p mimics (mimics-153), miR-153-3p inhibitor (inhi-153), and corresponding controls (mimics-NC, inhi-NC) were purchased from GenePharma. The above plasmids or siRNAs were transfected into cells using Lipofectamine 2000 (Invitrogen, Carlsbad, CA, USA). The transfection efficiency was verified by reverse transcription-quantitative polymerase chain reaction (RT-qPCR) or western blot 48 h later.

### Treatment of ferroptosis inhibitors

The cells were pre-treated with 5 µM ferrostatin-1 (SML0583, Sigma-Aldrich, Missouri, USA) or dimethyl sulfoxide (DMSO) (Solarbio, Beijing, China) for 24 h before irradiation.

### Irradiation

Irradiation at various doses was performed at room temperature using a 6-megavolt X-ray linear accelerator (Varian, EDGE, USA). The radiation conditions were as follows: treatment field of 40 × 40 cm, source-skin distance of 100 cm, and radiation dose rate of 5 Gy/min.

### Cell Counting Kit-8 (CCK-8) method

Cell viability was assessed using the CCK-8 assay. The cells (2000 cells/well) were seeded in 96-well plates. At 24, 48, and 72 h after irradiation, 10 µL of CCK-8 solution (02432300, Cellor Lab, China) was added for further 3-h incubation. The optical density (OD) values were measured at 450 nm using a microplate reader (Multiskan™ FC, 51119180ET, Thermo Fisher Scientific, USA). Additionally, a blank background group containing only DMEM was set to eliminate the OD value of the medium. The cell proliferation rate was calculated using the following formula: cell proliferation rate (%) ═ (OD treatment group - OD blank)/(OD control group - OD blank) × 100%.

### Colony formation assay

Cell survival after radiation was defined as the ability of cells to maintain clonogenic capacity and subsequently form colonies. Cells were counted and seeded in 6-well plates at 500 cells/well. Cells were exposed to the indicated doses of radiation and incubated at 37 ^∘^C for 12–14 days. Colonies were stained with crystal violet and manually counted, with colonies containing ≥ 50 cells recorded.

### Immunofluorescence

Cells were subjected to fixation with 4% paraformaldehyde and permeabilization with 1% Triton X-100. After blocking, the cells were incubated with the phosphorylated H2A histone family member x (γ-H2AX) antibody (A700-053, Thermo Fisher Scientific, USA) for 2 h or IgG antibody (ab150079, Abcam) for 1 h, followed by staining with 4′,6-diamidino-2-phenylindole (DAPI) (Thermo Fisher Scientific) for 5 min at 37 ^∘^C. Finally, cell observation was performed using a fluorescence microscope (Olympus, Japan).

### Fe^2+^, reactive oxygen species (ROS), and glutathione (GSH) assays

Fe^2+^ levels in cells were detected using the Iron Ion Colorimetric Assay Kit (E1042, Applygen Technologies, Beijing, China). ROS levels were measured by adding a 20 µM cell permeabilization probe, 2′,7′-dichlorodihydrofluorescein diacetate (DCFH-DA) (Sigma). After incubation for 30 min, the fluorescence intensity at 485 nm excitation and 530 nm emission wavelengths was measured using a fluorescence spectrometer. For GSH detection, a commercially available GSH assay kit (A006-1-1, Nanjing, China) was used. The absorbance at 420 nm was measured to calculate the GSH content.

### Quantification of m6A

m6A RNA methylation was detected using the m6A RNA Methylation Assay Kit (ab185912, Abcam). The absorbance at 450 nm was measured, and the percentage of m6A relative to total RNA (400 ng) in each group was calculated.

### Methylated RNA immunoprecipitation (MeRIP)

MeRIP assays for gene m6A modifications were performed using the Magna MeRIP Kit (CR203146, Millipore, MA, USA). Briefly, cells were washed twice with ice-cold PBS and then collected by centrifugation (1500 g, 4 ^∘^C, 5 min). After removal of the supernatant, cells were mixed with 100 µL of RNA immunoprecipitation (RIP) lysis buffer and incubated on ice for 5 min. Anti-m6A antibody (ab208577, Abcam) was coated on magnetic beads, washed twice with RIP washing buffer, and resuspended in 900 µL of RIP immunoprecipitation buffer and 100 µL of cell lysate. After an overnight incubation at 4 ^∘^C, the beads were washed, and RNA enrichment was analyzed by RT-qPCR.

### RNA stability assay

The cells were seeded in 6-well plates and treated with actinomycin D (5 µg/mL, Sigma-Aldrich, St. Louis, MO, USA) for 0, 3, and 6 h. Total RNA was then extracted for RT-qPCR analysis.

### Nuclear/cytoplasmic fractionation

Cellular localization was performed using the PARIS kit (Invitrogen). Briefly, the nuclear and cytoplasmic fractions were separated and analyzed by RT-qPCR. U6 snRNA and glyceraldehyde 3-phosphate dehydrogenase (GAPDH) were used as positive controls for the nuclear and cytoplasmic fractions, respectively.

### RNA immunoprecipitation (RIP)

RIP analysis was performed using the Magna RIP kit (Millipore, Billerica, MA, USA). Cells were lysed in lysis buffer containing protease and ribonuclease inhibitors for 30 min on ice, followed by centrifugation (25,000 *g*, 4 ^∘^C, 5 min). The supernatant was then used as input (positive control). IgG (ab172730, Abcam), argonaute 2 (Ago2) (ab186733, Abcam), and protein A/G beads were added to the supernatant. After centrifugation at 4 ^∘^C, the samples were incubated overnight. The protein A/G-bead precipitates were washed three times, and the relative RNA in the precipitate, after isolation and purification, was verified by RT-qPCR.

### Dual-luciferase assay

The binding relationships between the competing endogenous RNA (ceRNA) networks were predicted using the Targetscan database (https://www.targetscan.org/vert_71/) [22] and the Starbase database (https://rnasysu.com/encori/index.php) [23]. The 3′-untranslated region (UTR) sequences of ENBL3P and KIAA1429 mRNA, containing miR-153-3p complementary sites, were cloned into pGL3-control luciferase reporter vectors (Promega, Madison, WI, USA), named ENBL3P-WT or KIAA1429-WT. The mutant sequences were named ENBL3P-MUT or KIAA1429-MUT. WT or MUT sequences were co-transfected into cells with miR-153-3p mimics or mimics-NC. 24 h after transfection, luciferase activity was assessed using the luciferase reporter system (Promega).

### RT-qPCR

Total RNA was extracted from cells using the TRIzol reagent (Invitrogen) and reverse transcribed into complementary DNA (cDNA) at 42 ^∘^C for 30 min using the PrimeScript™ RT kit (Takara). qPCR was performed using iTaq™ Universal SYBR^®^ Green Supermix (Bio-Rad) on an ABI 7500 instrument (Applied Biosystems). GAPDH or U6 [24] was used as an internal reference. Relative expression was calculated using the 2^−ΔΔCt^ method [25]. Primers are shown in Table 1.

**Table 1:** PCR primer sequences

**Name**	**Sequence (5′-3′)**
KIAA1429	F: CGAGCGCTGAGCAAAGTTCT
	R: TGGGGGTATGACTCGGACTT
LncRNA EBLN3P	F: GTCCAGTCTTTGAGGACCGA
	R: CCTATGCCCAGATCGTCCAA
GAPDH	F: GTCAAGGCTGAGAACGGGAA
	R: TCGCCCCACTTGATTTTGGA
miR-153-3p	F: GCGTCGATTGCATAGTCACAA
	R: AACTGGTGTCGTGGAGTCGG
U6	F: TCGCTTCGGCAGCACATATACT
	R: GCTTCACGAATTTGCGTGTCATC

PCR: Polymerase chain reaction; KIAA1429: Vir like m6A methyltransferase associated; LncRNA: Long non-coding RNAs; EBLN3P: Endogenous Bornavirus like nucleoprotein 3, pseudogene; GAPDH: Glyceraldehyde-3-phosphate dehydrogenase; miR: Micro RNA.

### Western blot

Proteins were collected from cells using the RIPA buffer (Beyotime, Shanghai, China). Protein concentration was determined by the bicinchoninic acid method (Beyotime). Proteins (40 µg/lane) were then separated by 10% sodium dodecyl sulfate–polyacrylamide gel electrophoresis (SDS-PAGE) (Beyotime) and transferred to polyvinylidene difluoride (PVDF) membranes (EMD Millipore). The PVDF membranes were blocked with 5% skim milk powder for 1.5 h at room temperature. The membranes were then incubated with primary antibodies against KIAA1429 (1:1000, ab271136, Abcam), solute carrier family 7-member 11(SLC7A11) (1:1000, ab307601, Abcam), glutathione peroxidase 4 (GPX4) (1:1000, ab125066, Abcam), acyl coenzyme a synthetase long chain 4 (ACSL4) (1:10,000, ab155282, Abcam), and GAPDH (1:2500, ab9485, Abcam) overnight at 4 ^∘^C. The next day, membranes were washed three times with PBS containing 0.2% Tween-20 (PBST) and treated with a secondary antibody (1:2000, ab205718, Abcam) for 1.5 h at room temperature. The immunoreactive bands were then washed with PBST. Finally, the signals were detected and visualized using an enhanced chemiluminescence assay (EMD Millipore) and an Odyssey infrared imaging system (LI-COR Biosciences). GAPDH was used as an internal reference. ImageJ software (version 1.52r; NIH) was utilized to semiquantify the intensity of the bands.

**Figure 1.**
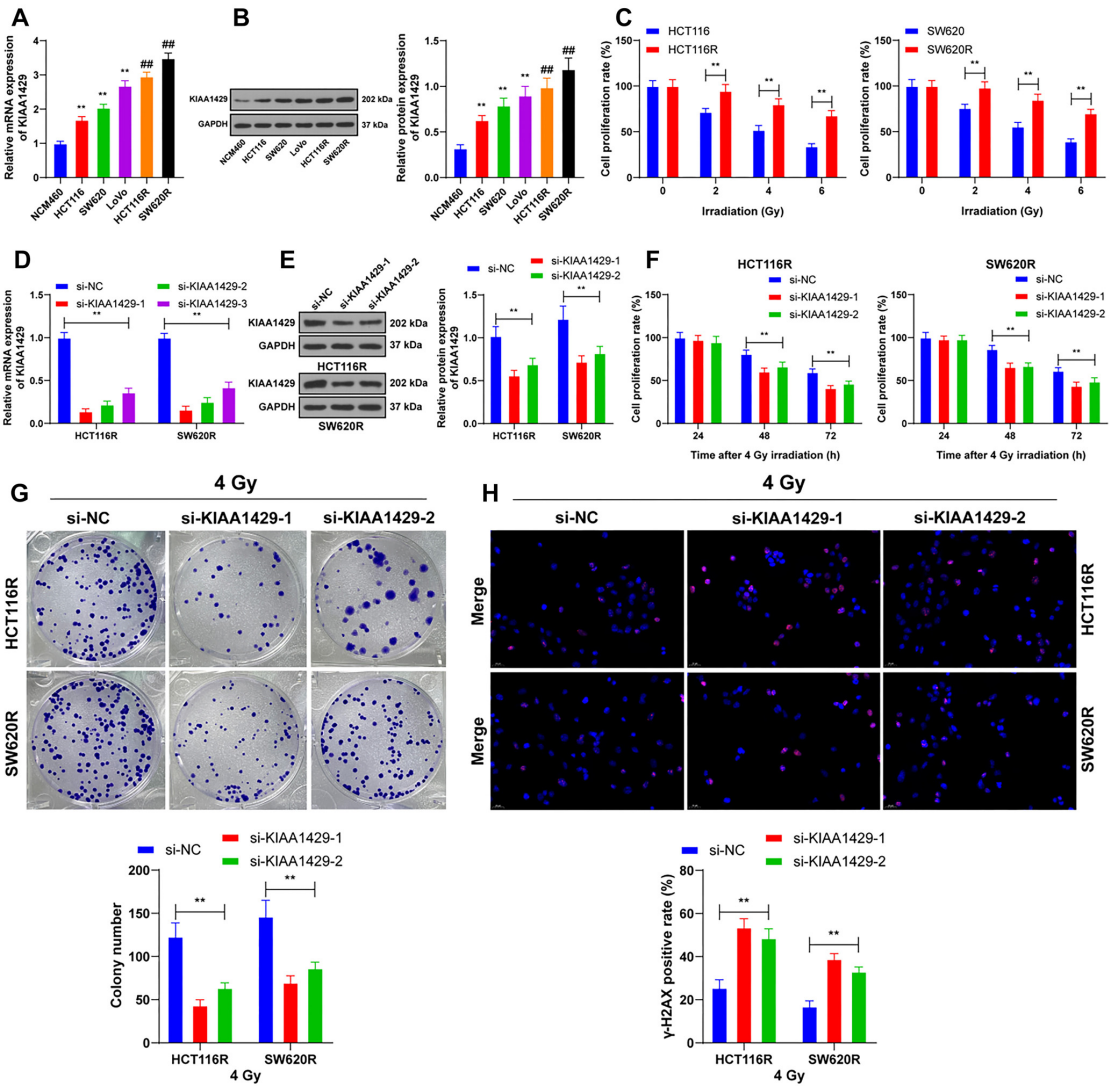
**KIAA1429 increases radioresistance in CRC cells.** (A and B) RT-qPCR and western blot analysis detecting the expression of KIAA1429 in cells. ^**^P < 0.01 compared with NCM460; ^##^*P* < 0.01 compared with parental cells; (C) Displaying the constructed radioresistant cell lines and the proliferation rate of cells detected by the CCK-8 assay at different radiation doses over 48 h. si-KIAA1429 was transfected into radioresistant cells, with si-NC used as a control; (D and E) Expression of KIAA1429 in the cells detected by RT-qPCR and western blot; (F) CCK-8 assay detecting the proliferation rate of cells at different times following 4 Gy of radiation; (G) Colony formation assay used to detect the clonogenic ability of the cells after 4 Gy of radiation; (H) Immunofluorescence analysis showcasing the positive rate of γ-H2AX in cells 48 h after 4 Gy of radiation. Three independent replicate tests were performed, and the data are expressed as mean ± standard deviation. One-way ANOVA was used for data comparisons among multiple groups in panels A and B; two-way ANOVA was used for data comparisons among multiple groups in panels C–H, and Tukey’s multiple comparisons test was used for all post-hoc tests. **P < 0.01. CRC: Colorectal cancer; RT-qPCR: Reverse transcription-quantitative polymerase chain reaction; CCK-8: Cell counting kit-8; γ-H2AX: Phosphorylated Histone H2AX; si-KIAA1429: KIAA1429 siRNA; si-NC: Negative control siRNA; ANOVA: Analysis of variance.

**Figure 2.**
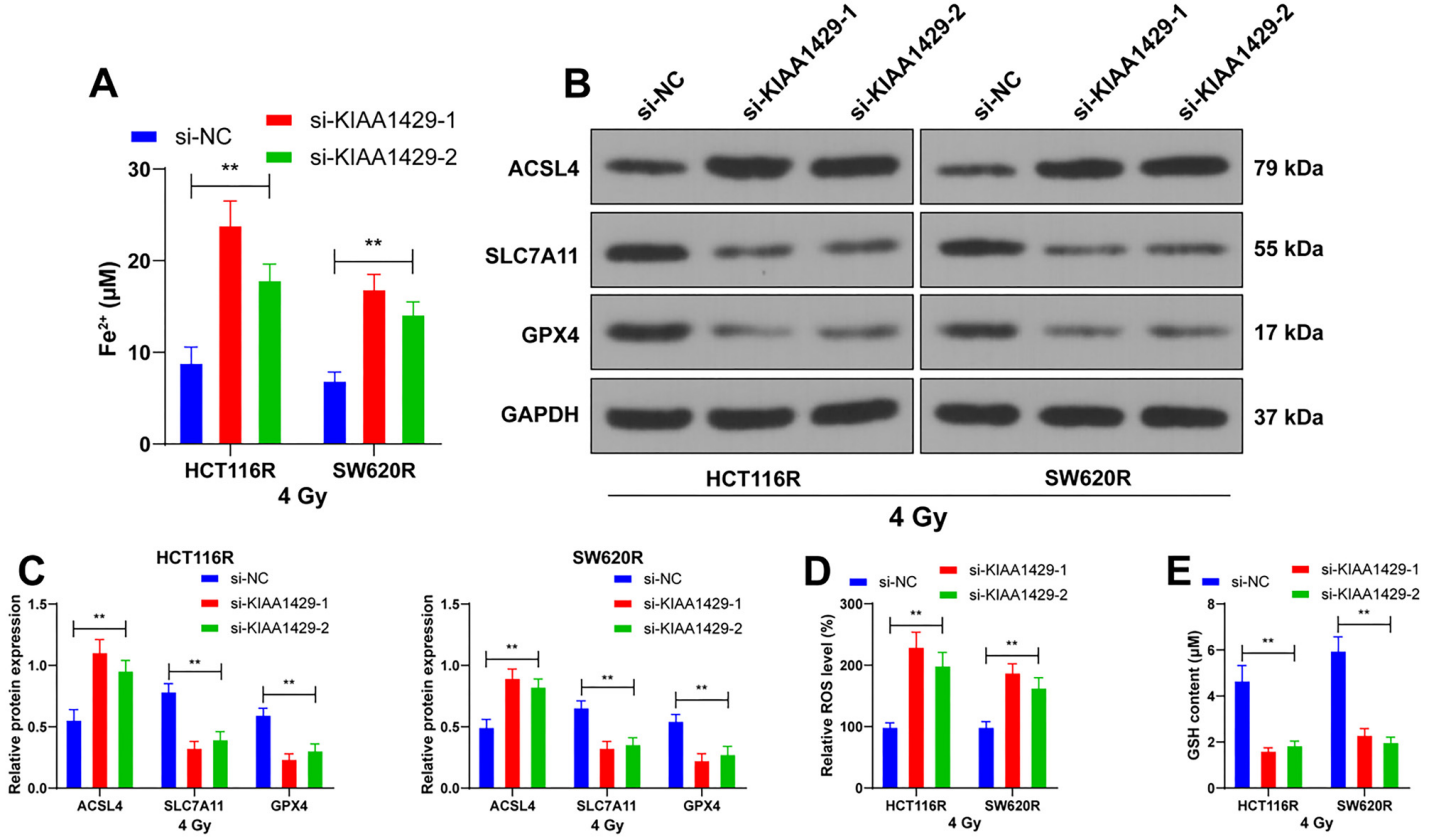
**KIAA1429 knockdown promotes ferroptosis in CRC cells.** The si-KIAA1429 was transfected into radioresistant cells, and si-NC was used as a control. (A) 48 h after 4 Gy radiation, the level of Fe^2+^ in cells with different transfections was detected; (B and C) Showcasing the expression of ferroptosis-related proteins ACSL4, SLC7A11, and GPX4 in cells with different transfections detected by western blot; (D) Displaying the level of ROS in cells with different transfections; (E) Showcasing the cellular changes in GSH levels in cells with different transfections. Three independent replicate assays were performed, and the data were expressed as mean ± standard deviation. Comparison of data among multiple groups was performed by two-way ANOVA, and Tukey’s test was used for all post-hoc tests. **P < 0.01. si-KIAA1429: KIAA1429 siRNA; si-NC: NC siRNA; ACSL4: Acyl Coenzyme A Synthetase Long Chain 4; GPX4: Glutathione peroxidase 4; SLC7A11: Solute carrier family 7-member 11; ROS: Reactive oxygen species; GSH: Glutathione; ANOVA: Analysis of variance; CRC: Colorectal cancer.

### Statistical analysis

All data were statistically analyzed and graphed using SPSS 21.0 statistical software (IBM, NY, USA) and GraphPad Prism 8.0 software (GraphPad Software Inc., San Diego, CA, USA). Normality and chi-square tests were first performed to confirm normal distribution and homogeneity of variance. The *t*-test was used for data comparison between two groups for measurement data; one-way or two-way analysis of variance (ANOVA) was used for data comparison among multiple groups, with Tukey’s test applied for post-hoc analyses. *P* values were obtained from two-sided tests, with *P* < 0.05 indicating statistical significance and *P* < 0.01 indicating extreme significance.

**Figure 3.**
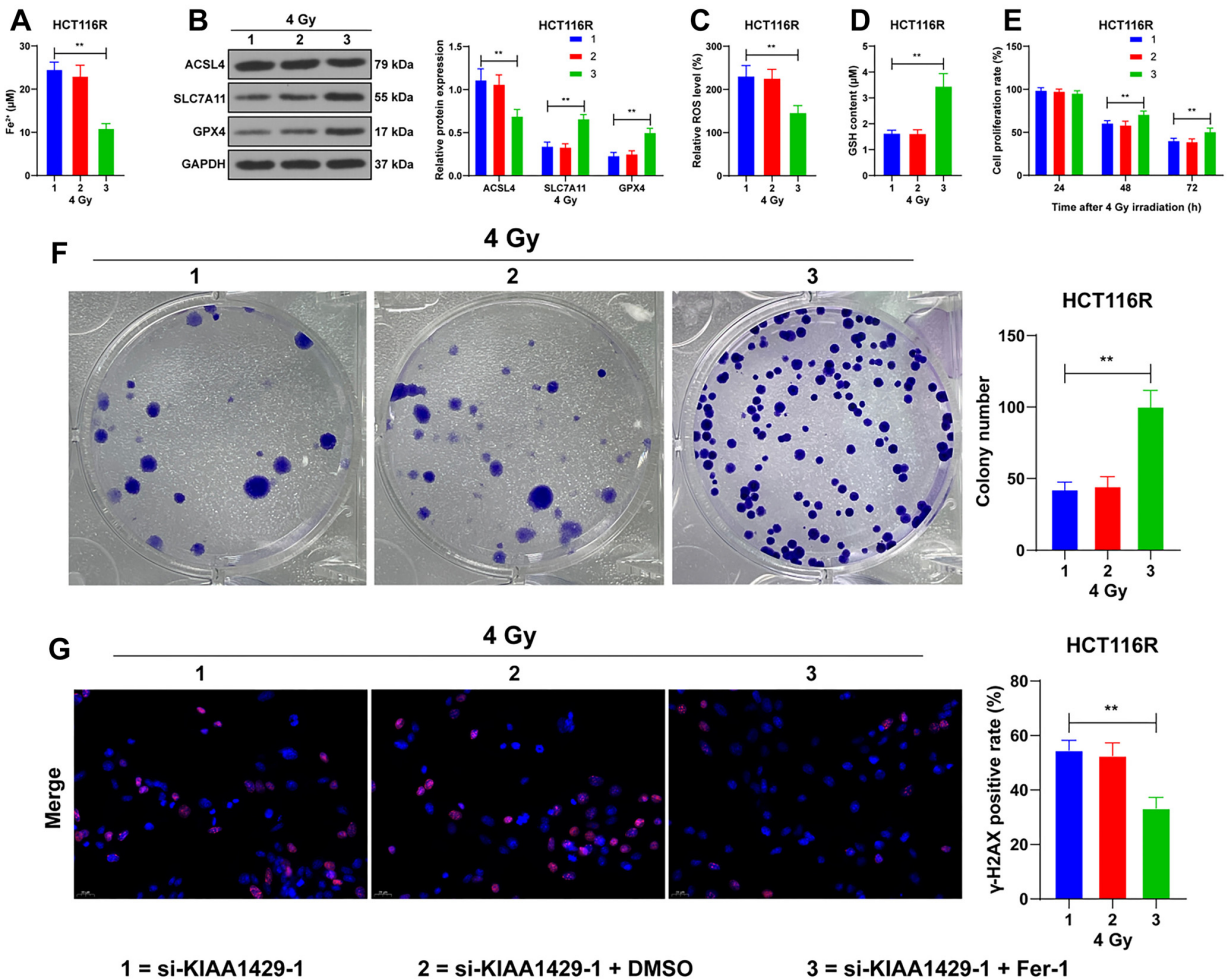
**KIAA1429 increases radioresistance in CRC cells by inhibiting ferroptosis.** HCT116R cells were treated with ferrostatin-1 (Fer-1), with DMSO treatment used as a control. (A) Displaying the level of Fe^2+^ detected in different treatments after 48 h of 4 Gy radiation; (B) Showcasing the expression of ferroptosis-related proteins ACSL4, SLC7A11, and GPX4 in cells with different treatments, detected by western blot; (C) Displaying the level of ROS in cells with different treatments; (D) Showcasing changes in GSH content in cells with different treatments; (E) CCK-8 assay detecting the proliferation rate of cells at different times after 4 Gy radiation; (F) Colony formation assay detecting the clonogenic ability of cells after 4 Gy radiation; (G) Immunofluorescence analysis displaying the positive rate of γ-H2AX in cells after 48 h of 4 Gy radiation. Three independent replicate tests were performed, and the data were expressed as mean ± standard deviation; one-way ANOVA was used to compare the data among multiple groups in panels A, C, D and F; two-way ANOVA was used to compare the data among multiple groups in panels B and E, and Tukey’s test was used in all post-hoc tests. *P* < 0.01. Fer-1: Ferrostatin-1; DMSO: Dimethyl sulfoxide; si-KIAA1429: KIAA1429 siRNA; ACSL4: Acyl Coenzyme A Synthetase Long Chain 4; GPX4: Glutathione peroxidase 4; SLC7A11: Solute carrier family 7-member 11; ROS: Reactive oxygen species; GSH: Glutathione; CCK-8: Cell counting kit-8; γ-H2AX: Histone H2AX; ANOVA: Analysis of variance; CRC: Colorectal cancer.

## Results

### KIAA1429 increases radioresistance in CRC cells

KIAA1429 is highly expressed in CRC, but its effect on cancer cell radioresistance remains unclear. We examined KIAA1429 expression, and the results showed a significant increase in KIAA1429 expression in CRC cells (*P* < 0.01; Figure 1A and 1B). We selected two cell lines with relatively low KIAA1429 expression to establish the radioresistant cell lines HCT116R and SW620R. Compared with the parental cells, the radioresistant cells exhibited a higher proliferation rate (*P* < 0.01; Figure 1C) and higher expression of KIAA1429 (*P* < 0.01; Figure 1A and 1B). To verify the role of KIAA1429 in CRC cell radioresistance, we downregulated KIAA1429 expression in these cells (*P* < 0.01; Figure 1D and 1E). The si-KIAA1429-1 and si-KIAA1429-2 constructs, which showed better intervention efficiency, were selected for subsequent experiments. Parental cells with a survival rate close to 50% were treated with 4 Gy radiation. Cells with reduced KIAA1429 expression exhibited a slower proliferation rate (*P* < 0.01; Figure 1F) and a reduced number of clonogenic cells (*P* < 0.01; Figure 1G). The positivity of γ-H2AX was notably increased after the knockdown of KIAA1429 (*P* < 0.01; Figure 1H). These results suggest that KIAA1429 promotes radioresistance in CRC cells.

### KIAA1429 knockdown promotes ferroptosis in CRC cells

Numerous studies have reported that ferroptosis is associated with radioresistance in cancer cells. We explored the effect of KIAA1429 on ferroptosis. After KIAA1429 knockdown, 4 Gy radiation resulted in increased levels of Fe^2+^ in HCT116R and SW620R cells (*P* < 0.01; Figure 2A). In addition, the expression of the ferroptosis-associated protein ACSL4 was enhanced, while SLC7A11 and GPX4 levels decreased following KIAA1429 knockdown (*P* < 0.01; Figure 2B and 2C). Downregulation of KIAA1429 also led to increased ROS levels and decreased GSH content (*P* < 0.01; Figure 2D and 2E). These findings indicate that KIAA1429 knockdown promotes ferroptosis in CRC cells.

### KIAA1429 increases radioresistance in CRC cells by inhibiting ferroptosis

To validate the role of ferroptosis in the modulation of radioresistance by KIAA1429, we used ferrostatin-1 (Fer-1), a ferroptosis inhibitor. As shown in Figure 3A and 3B, treatment with Fer-1 and 4 Gy radiation led to decreased levels of Fe^2+^ and ACSL4, along with increased levels of SLC7A11 and GPX4 in HCT116R cells (*P* < 0.01). Additionally, Fer-1 treatment resulted in decreased ROS levels and increased GSH content (*P* < 0.01; Figure 3C and 3D). After reducing ferroptosis, the proliferation rate of the cells increased (*P* < 0.01; Figure 3E), the number of clonogenic cells increased (*P* < 0.01; Figure 3F), and the positivity of γ-H2AX was markedly reduced (*P* < 0.01; Figure 3G). In summary, KIAA1429 increases the radioresistance of CRC cells by inhibiting ferroptosis.

### KIAA1429-mediated m6A modification upregulates LncRNA EBLN3P expression

**Figure 4.**
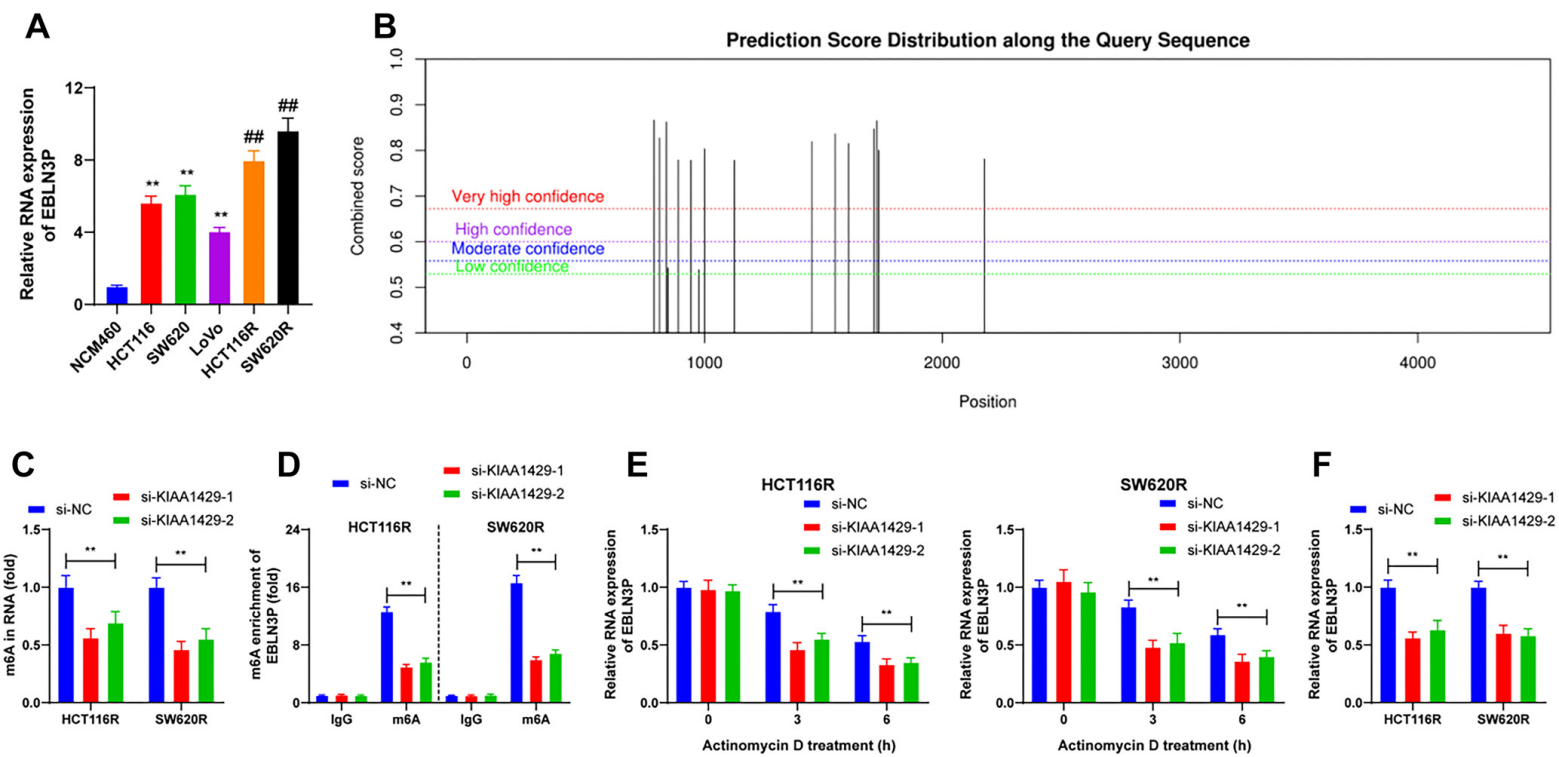
**KIAA1429-mediated m6A modification upregulates the expression of LncRNA EBLN3P.** (A) Showcasing the RT-qPCR detection of EBLN3P expression in cells of each group. **P < 0.01 compared with NCM460; ^##^*P* < 0.01 compared with parental cells; (B) m6A online tool prediction of m6A modification sites on EBLN3P; (C) m6A quantification analysis of m6A enrichment in cells with different transfections; (D) MeRIP analysis of the m6A level in EBLN3P RNA in cells with different transfections; (E) RT-qPCR detection of the RNA stability of EBLN3P in cells with different transfections; (F) RT-qPCR detection of EBLN3P expression in cells with different transfections. Three independent replicate assays were performed, and the data were expressed as mean ± standard deviation. One-way ANOVA was used to compare the data among multiple groups in panel A, two-way ANOVA was used to compare the data among multiple groups in panels C–F, and Tukey’s test was used in all post-hoc tests. **P < 0.01. RT-qPCR: Reverse transcription-quantitative polymerase chain reaction; m6A: N6-methyladenosine; MeRIP: RNA methylation immunoprecipitation; si-KIAA1429: KIAA1429 siRNA; si-NC: Negative control siRNA; ANOVA: Analysis of variance.

KIAA1429 is a major m6A methyltransferase, and EBLN3P expression is known to be increased in CRC [16]. Our results corroborated this, showing elevated EBLN3P expression in CRC cells, particularly in radioresistant cells (*P* < 0.01; Figure 4A). Using the m6A online tool (http://www.cuilab.cn/sramp/), a sequence-based m6A modification site predictor, we identified m6A sites on the EBLN3P sequence (Figure 4B). We speculated that EBLN3P is downstream of KIAA1429. Quantitative m6A analysis revealed that the m6A enrichment level was downregulated in KIAA1429 low-expressing cells (*P* < 0.01; Figure 4C). Further analysis indicated that m6A levels were upregulated in EBLN3P RNA but decreased following KIAA1429 knockdown (*P* < 0.01; Figure 4D). RNA stability assays demonstrated that KIAA1429 knockdown led to reduced stability of EBLN3P (*P* < 0.01; Figure 4E). Correspondingly, EBLN3P levels were reduced after KIAA1429 knockdown (*P* < 0.01; Figure 4F). Collectively, these findings suggest that KIAA1429 enhances LncRNA EBLN3P expression in an m6A-dependent manner.

### Overexpression of EBLN3P inhibits ferroptosis to increase radioresistance in CRC cells

**Figure 5.**
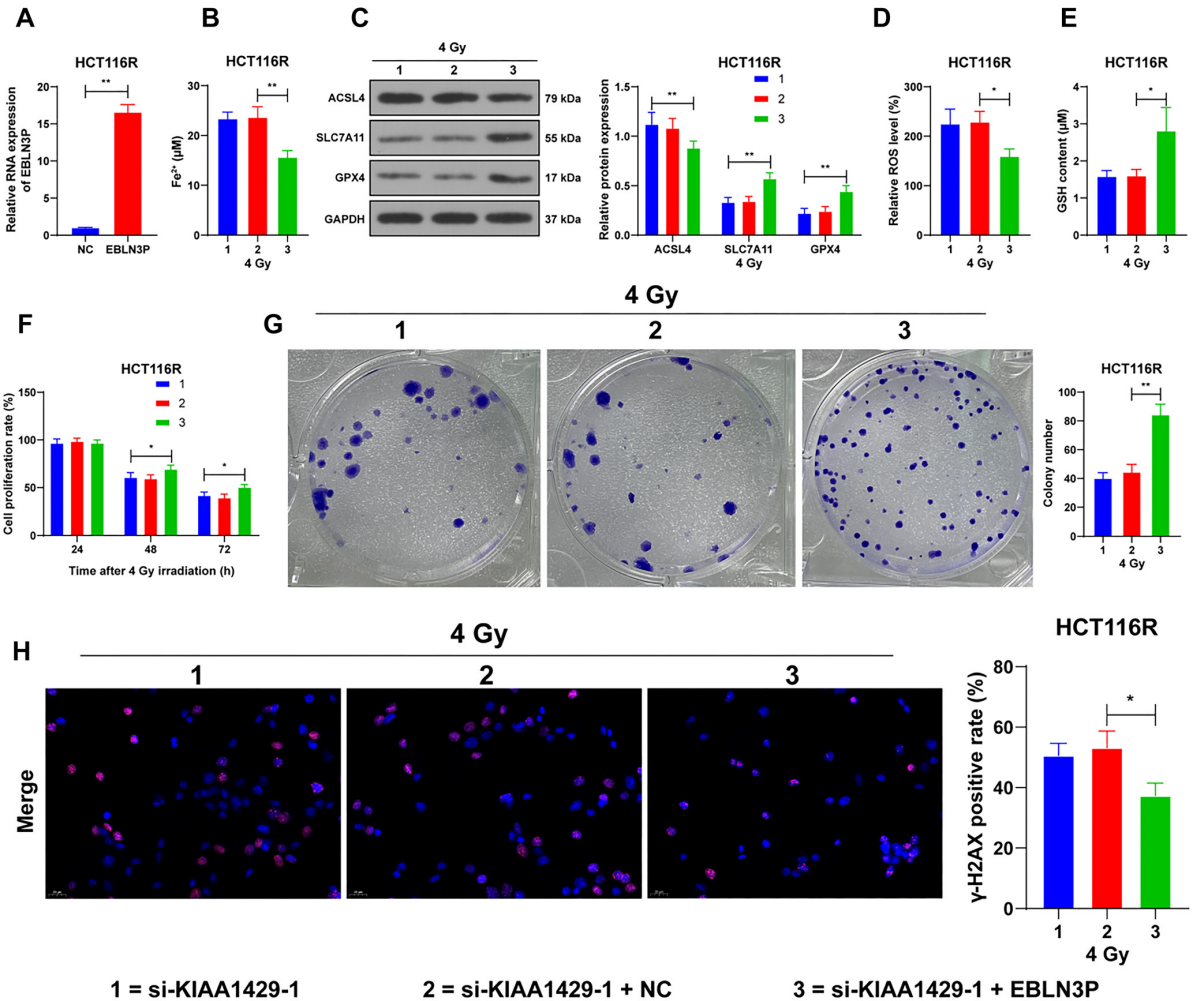
**Overexpression of EBLN3P inhibits ferroptosis to increase radioresistance in CRC cells.** HCT116R cells were treated with ferrostatin-1 (Fer-1), with DMSO treatment used as a control. (A) Displaying the RT-qPCR detection of EBLN3P expression in cells following 48 h of 4 Gy radiation; (B) Showcasing the level of Fe^2+^ in cells after the overexpression of EBLN3P; (C) Western blot analysis displaying the expression of ferroptosis-related proteins ACSL4, SLC7A11, and GPX4 in cells after overexpression of EBLN3P; (D) Showcasing the level of ROS; (E) Illustrating the changes in GSH content in cells after the overexpression of EBLN3P; (F) CCK-8 assay displaying the proliferation rate of cells at different times after 4 Gy radiation; (G) Colony formation assay detecting the clonogenic ability of cells after 4 Gy radiation; (H) Immunofluorescence analysis detecting the positive rate of γ-H2AX in cells after 48 h of 4 Gy radiation. Three independent replicate tests were performed, and the data were expressed as mean ± standard deviation. The *t*-test was used to compare the data in panel A; one-way ANOVA was used to compare the data among multiple groups in panels B, D, E, G, and H; two-way ANOVA was used to compare the data among multiple groups in panel C and F, and Tukey’s test was used in all post-hoc tests. *P < 0.05; **P < 0.01. EBLN3P: EBLN3P overexpression vectors; NC: Empty vector; DMSO: Dimethyl sulfoxide; RT-qPCR: Reverse transcription-quantitative polymerase chain reaction; ACSL4: Acyl coenzyme a synthetase long chain 4; GPX4: Glutathione peroxidase 4; SLC7A11: Solute carrier family 7-member 11; ROS: Reactive oxygen species; GSH: Glutathione; CCK-8: Cell Counting Kit-8; γ-H2AX: Histone H2AX; ANOVA: Analysis of variance; CRC: Colorectal cancer.

Next, we upregulated EBLN3P expression in HCT116R cells (*P* < 0.01; Figure 5A) co-treated with si-KIAA1429-1. The level of ferroptosis was reduced in cells overexpressing EBLN3P after 4 Gy radiation (*P* < 0.05; Figure 5B–5E). Additionally, the proliferation rate of cells increased following EBLN3P overexpression (*P* < 0.05; Figure 5F), the number of clonogenic cells was enhanced (*P* < 0.01; Figure 5G), and the positivity of γ-H2AX was markedly reduced (*P* < 0.05; Figure 5H). Altogether, these results indicate that overexpression of EBLN3P inhibits ferroptosis, thereby increasing radioresistance in CRC cells.

### LncRNA EBLN3P competitively binds to miR-153-3p through the ceRNA network to promote KIAA1429 expression

**Figure 6.**
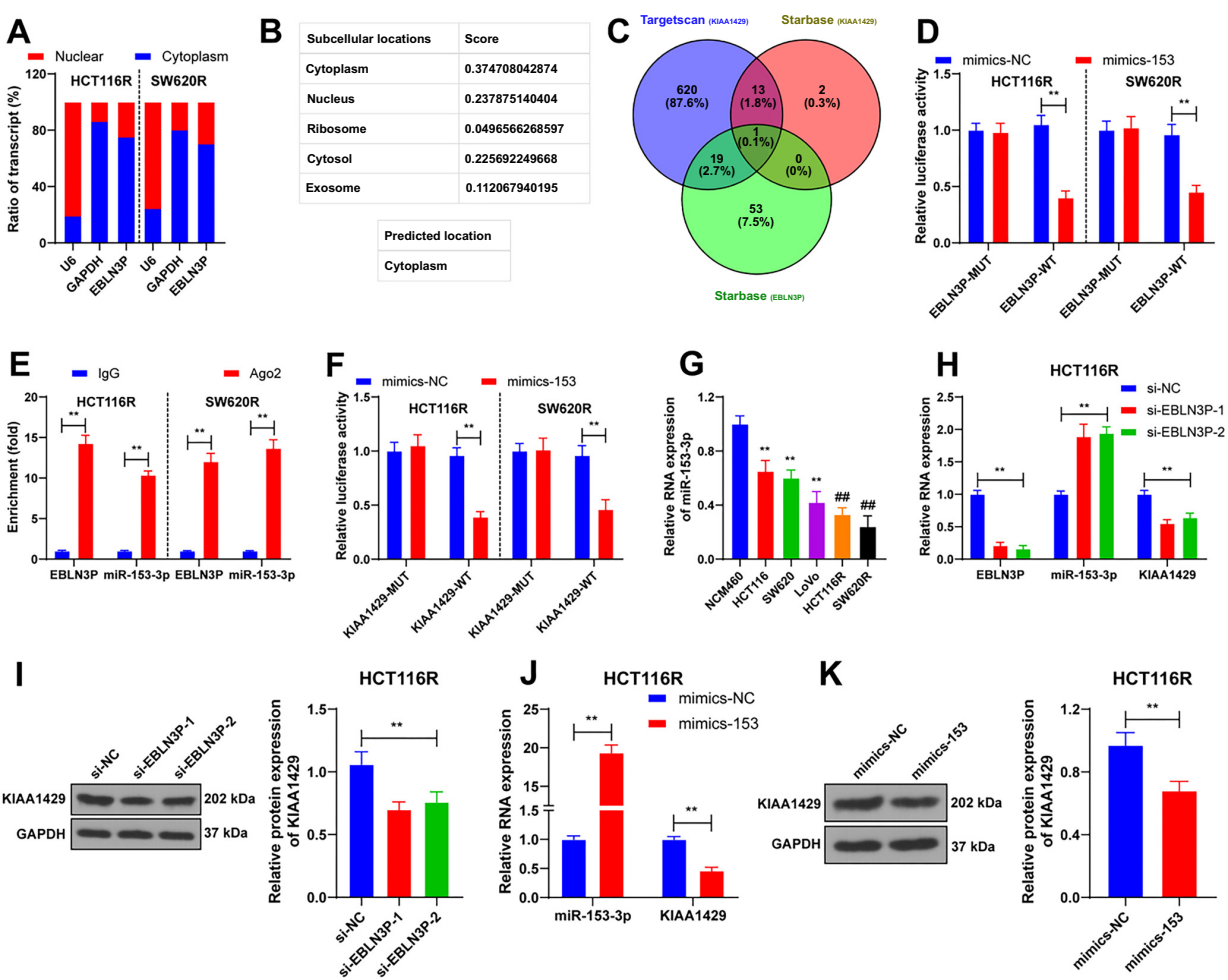
**LncRNA EBLN3P competitively binds to miR-153-3p through the ceRNA network to promote KIAA1429 expression.** (A) Nuclear/cytoplasmic fractionation detecting the subcellular localization of EBLN3P in CRC cells; (B) Database prediction of the subcellular localization of EBLN3P; (C) TargetScan and StarBase databases predicting the ceRNA mechanism of EBLN3P; (D–F) Dual-luciferase and RIP experiments validating the ceRNA mechanism of EBLN3P in CRC cells; (G) RT-qPCR detecting the miR-153-3p expression in cells of each group. **P < 0.01 compared with NCM460; ^##^*P* < 0.01 compared with parental cells; (H) RT-qPCR detecting the EBLN3P, miR-153-3p, and KIAA1429 expression after transfection of si-EBLN3P; (I) Western blot analysis showcasing the KIAA1429 expression after transfection of si-EBLN3P; (J) RT-qPCR displaying the miR-153-3p and KIAA1429 expression after transfection of mimics-153; (K) Western blot analysis detecting the KIAA1429 expression after transfection of mimics-153. Three independent replicate assays were performed, and the data were expressed as mean ± standard deviation. A *t*-test was used to compare the data between the two groups in panel K; one-way ANOVA was used to compare the data among multiple groups in panels G and I; two-way ANOVA was used to compare the data among multiple groups in panels D–F, H, and J, and Tukey’s test was used in all post-hoc tests. **P < 0.01. RIP: RNA immunoprecipitation; ceRNA: Competing endogenous RNA; RT-qPCR: Rreverse transcription-quantitative polymerase chain reaction; si-EBLN3P: LncRNA EBLN3P siRNA; si-NC: NC siRNA; mimics-153: MiR-153-3p mimics; mimics-NC: NC mimics; ANOVA: Analysis of variance; CRC: Colorectal cancer.

In radioresistant cells, subcellular analysis revealed that EBLN3P was predominantly located in the cytoplasm (Figure 6A), consistent with predictions from an online database (http://www.csbio.sjtu.edu.cn/bioinf/lncLocator/?tdsourcetag=s_pcqq_aiomsg) (Figure 6B). This finding led us to explore the mechanism of EBLN3P-mediated ceRNA. By analyzing the intersection between EBLN3P and KIAA1429, we identified miR-153-3p as the relevant miRNA (Figure 6C). A luciferase assay demonstrated a close association between miR-153-3p and EBLN3P (*P* < 0.01; Figure 6D). Ago2-RIP experiments further confirmed that miR-153-3p interacts with EBLN3P at the molecular level (*P* < 0.01; Figure 6E). Additionally, miR-153-3p was found to be closely associated with KIAA1429 mRNA 3′-UTR (*P* < 0.01; Figure 6F). miR-153-3p expression was reduced in CRC cells (*P* < 0.01; Figure 6G). Downregulation of EBLN3P promoted miR-153-3p expression and inhibited KIAA1429 expression (*P* < 0.01; Figure 6H and 6I), while overexpression of miR-153-3p also inhibited KIAA1429 expression (*P* < 0.01; Figure 6J and 6K). Taken together, these findings suggest that lncRNA EBLN3P competitively binds to miR-153-3p through the ceRNA network to promote KIAA1429 expression.

### Downregulation of miR-153-3p represses ferroptosis to increase radioresistance in CRC cells

**Figure 7.**
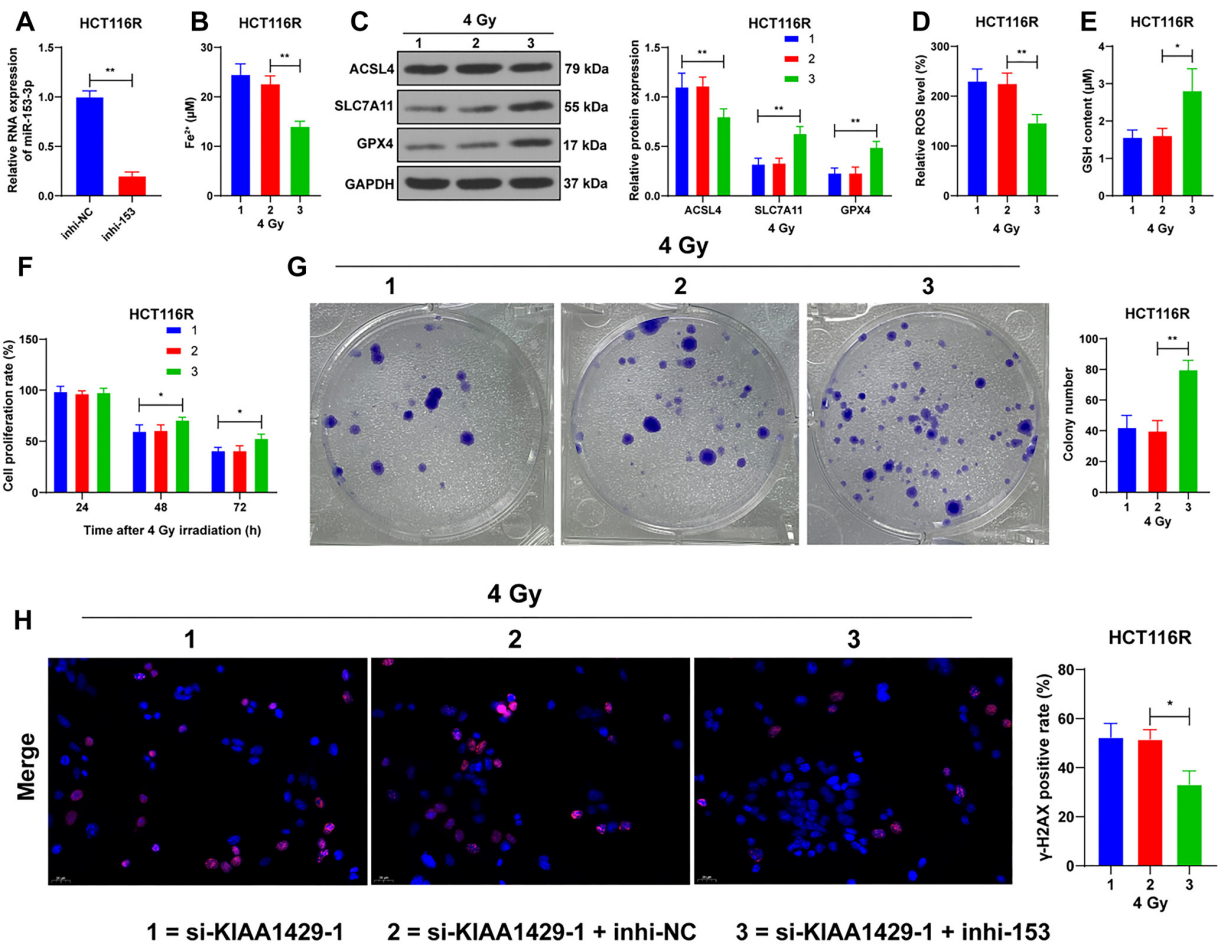
**Downregulation of miR-153-3p represses ferroptosis to increase radioresistance in CRC cells.** Inhi-153 was transfected into HCT116R cells, with inhi-NC as a control. (A) RT-qPCR detecting miR-153-3p expression in cells 48 h after 4-Gy radiation; (B) Fe^2+^ levels in cells after inhi-153 transfection; (C) Western blot analysis detecting the expression of ferroptosis-related proteins ACSL4, SLC7A11, and GPX4; (D) Showcasing the ROS levels in cells after inhi-153 transfection; (E) Displaying the changes in GSH content in cells after inhi-153 transfection; (F) CCK-8 assay detecting the proliferation rate of cells at different times after 4-Gy radiation; (G) Colony formation assay detecting the clonogenic ability of cells after 4-Gy radiation; (H) Immunofluorescence analysis detecting γ-H2AX positivity in cells 48 h after 4-Gy radiation. Three independent replicate tests were performed, and the data were expressed as the mean ± standard deviation. A *t*-test was used to compare the data between two groups in panel A; one-way ANOVA was used to compare the data among multiple groups in panels B, D, E, G, H; two-way ANOVA was used to compare the data among multiple groups in panels C and F, and post hoc tests were performed using Tukey’s test. *P < 0.05; **P < 0.01. inhi-153: miR-153-3p inhibitor; inhi-NC: NC inhibitor; RT-qPCR: Reverse transcription-quantitative polymerase chain reaction; ACSL4: Acyl Coenzyme A Synthetase Long Chain 4; GPX4: Glutathione peroxidase 4; SLC7A11: Solute carrier family 7-member 11; ROS: Reactive oxygen species; GSH: Glutathione; CCK-8: Cell Counting Kit-8; γ-H2AX: Histone H2AX; ANOVA: Analysis of variance; CRC: Colorectal cancer.

Finally, we decreased miR-153-3p expression in HCT116R cells (*P* < 0.01; Figure 7A) co-treated with si-KIAA1429-1. After 4 Gy radiation, miR-153-3p downregulation resulted in reduced ferroptosis in the cells (*P* < 0.05; Figure 7B–7E), increased proliferation (*P* < 0.05; Figure 7F), a higher number of clonogenic cells (*P* < 0.01; Figure 7G), and significantly decreased positivity of γ-H2AX (*P* < 0.05; Figure 7H). In summary, miR-153-3p downregulation inhibited ferroptosis, thereby increasing radioresistance in CRC cells.

## Discussion

**Figure 8.**
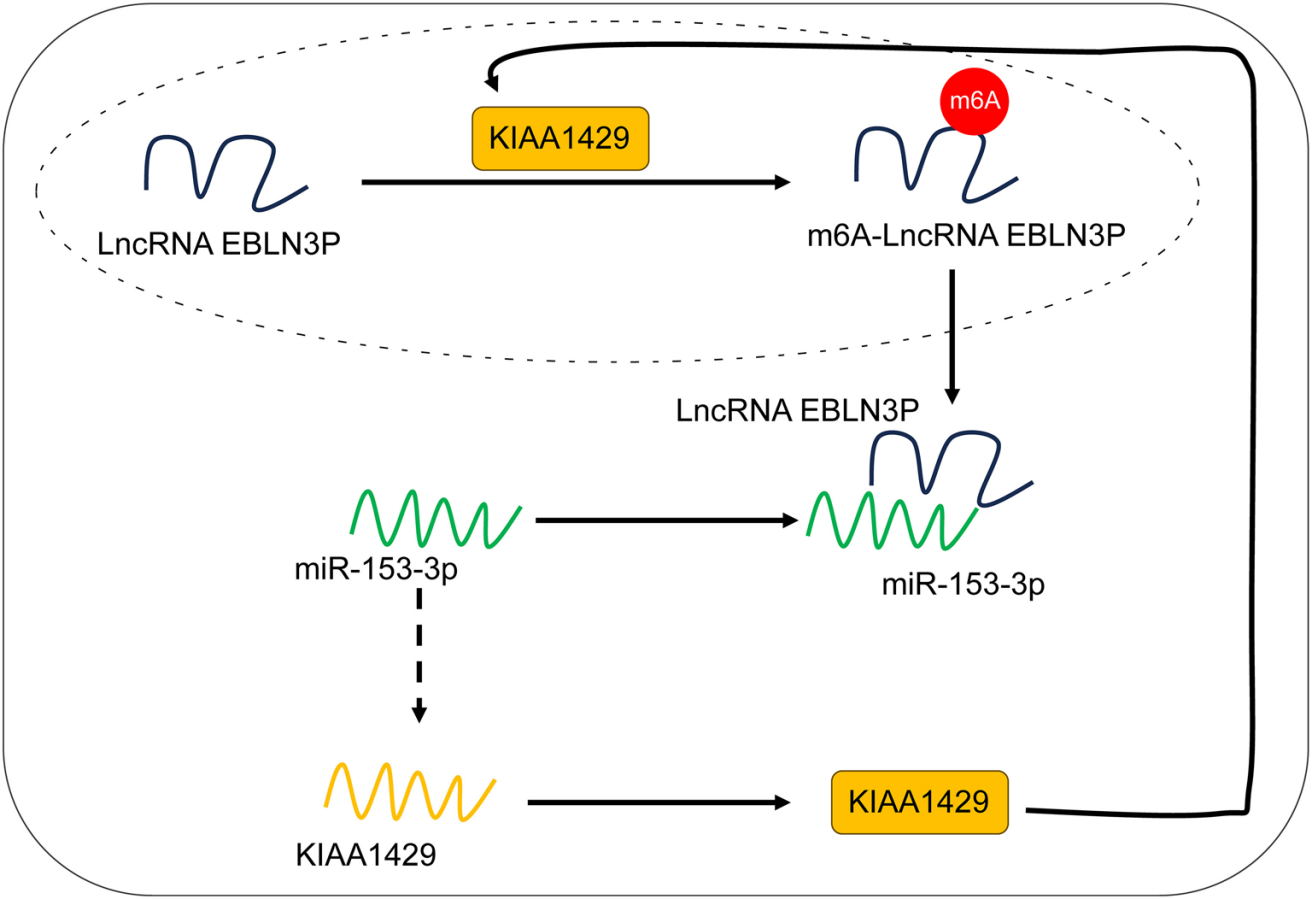
**KIAA1429/LncRNA EBLN3P/miR-153-3p feedback loop increases radioresistance in colorectal cancer cells by decreasing ferroptosis.** LncRNA: Long non-coding RNA; EBLN3P: Endogenous bornavirus-like nucleoprotein; miR: MicroRNA.

Radiotherapy is frequently used to treat CRC, yet tumor resistance to radiotherapy remains a major challenge [26]. Following radiotherapy, cancer cells often exhibit mitochondrial morphological alterations characteristic of ferroptosis, and ferroptosis agonists can enhance the radiation efficacy of tumor models [27, 28]. KIAA1429 has been associated with ferroptosis in multiple cancer cell types, except CRC [12, 29]. This study investigated the role of KIAA1429 in CRC cell ferroptosis and radioresistance, as well as the underlying mechanisms. Our findings revealed that KIAA1429-mediated m6A modification upregulates LncRNA EBLN3P expression, and in turn, lncRNA EBLN3P increases KIAA1429 expression by competitively binding to miR-153-3p, thereby reducing ferroptosis and enhancing the radioresistance of CRC cells (Figure 8).

KIAA1429 is significantly overexpressed in CRC tissues, and CRC patients with higher KIAA1429 expression have shorter overall survival compared to those with lower expression [30]. Our results similarly demonstrated high expression of KIAA1429 in CRC cells. Additionally, our data showed that CRC cells with KIAA1429 downregulation exhibited slower growth and fewer clonogenic cells. The positive rate of γ-H2AX, a predictive marker in radiation oncology [31], was elevated in CRC cells following KIAA1429 knockdown, indicating that KIAA1429 enhances the radiotherapy resistance of CRC cells. Consistent with these findings, KIAA1429 has been shown to promote cancer cell resistance to gefitinib and accelerate tumorigenesis in lung adenocarcinoma [32]. Ferroptosis, an iron-dependent form of regulated cell death, is driven by an overload of lipid peroxides on cellular membranes [33]. Factors, such as SLC7A11, ROS, GSH, GPX4, Fe^2+^, and ACSL4, are involved in the regulation of ferroptosis [34]. Our results demonstrated that the levels of ACSL4 and ROS were increased, while the levels of SLC7A11, GPX4, and GSH were decreased in HCT116R and SW620R cells following KIAA1429 silencing, suggesting that KIAA1429 downregulation enhances ferroptosis in CRC cells. Certain cancer cells with acquired drug resistance have been shown to exert antitumor effects by inducing ferroptosis [35]. Similarly, our results indicated that ferroptosis inhibition mediated by Fer-1 contributed to CRC cell proliferation, colony growth, and reduced γ-H2AX positivity. Collectively, these findings suggest that KIAA1429 enhances CRC cell radioresistance by suppressing ferroptosis.

Researchers have identified that KIAA1429 regulates LncRNA POU6F2-AS1 to aggravate CRC progression through m6A modification [36]. LncRNA EBLN3P shows a significant elevation in CRC patients [16], which is consistent with our results. Additionally, we identified m6A sites on the EBLN3P sequence and observed that m6A enrichment declined in CRC cells with silenced KIAA1429. Furthermore, the downregulation of KIAA1429 led to reduced stability and expression levels of EBLN3P. These results suggest that KIAA1429 could stimulate EBLN3P expression in an m6A-dependent manner. In non-small cell lung cancer cells, EBLN3P can enhance ROS production, which plays a crucial role in regulating radioresistance [37]. Therefore, we investigated whether EBLN3P affects ferroptosis and radioresistance in CRC cells. Interestingly, following EBLN3P upregulation, CRC cells exhibited suppressed ferroptosis, rapid proliferation, increased colony formation, and a higher γ-H2AX positivity rate. Higher EBLN3P levels have been associated with methotrexate resistance, and downregulation of EBLN3P decreased methotrexate resistance in osteosarcoma cells [38]. EBLN3P expression is higher in lung cancer tissues and is reduced by carbon ion irradiation [39]. There have been no reports on the regulation of LncRNA EBLN3P in ferroptosis and radioresistance in CRC. Therefore, our study is the first to highlight that lncRNA EBLN3P could enhance the radioresistance of CRC cells by suppressing ferroptosis.

The interaction between lncRNA and miRNA is involved in CRC pathogenesis [40]. LncRNA EBLN3P regulates UHMK1 expression by sponging miR-323a-3p, thereby contributing to CRC development [16]. miR-153 promotes cellular invasion in the progression of CRC, and its interaction with small nucleolar RNA host gene 17 (*SNHG17*)-collagen type XI alpha 1 (COL11A1)/insulin-like growth factor-binding protein 3 (IGFBP3)/krüppel-like factor 6 (KLF6) or with taurine upregulated gene 1 (*TUG1*)-death-associated protein kinase 1 (DAPK1)/aryl hydrocarbon receptor nuclear translocator 2 (ARNT2)/kallikrein-related peptidase 3 (KLK3)/phospholipase D1 (PLD1)/SMAD family member 2 (SMAD2) may play a role in early-stage colon adenocarcinoma [41]. In CRC patients, miR-153-5p is negatively correlated with LINC00511 [42]. Our results suggested that miR-153-3p is poorly expressed in CRC cells, and downregulation of EBLN3P increased miR-153-3p expression while suppressing KIAA1429 expression. This indicates that lncRNA EBLN3P can enhance KIAA1429 expression by competitively binding to miR-153-3p through the ceRNA network. There is limited research on the role of miR-153-3p in ferroptosis. Only one previous study identified the involvement of miR-153 in breast cancer ferroptosis [43]. Additionally, miR-153-3p has been shown to enhance cell radiosensitivity in human glioma by targeting BCL2 [44]. Our findings further demonstrated that miR-153-3p downregulation can increase the radioresistance of CRC cells by inhibiting ferroptosis.

## Conclusion

In summary, our results demonstrate that KIAA1429-mediated m6A modification upregulates lncRNA EBLN3P, while lncRNA EBLN3P, in turn, elevates KIAA1429 expression by competitively binding to miR-153-3p through the ceRNA network. This interaction reduces ferroptosis and enhances the radioresistance of CRC cells. However, several limitations should be addressed in future studies. Firstly, the proposed mechanism was verified only at the cellular level, lacking animal experiments to further validate the findings, and clinical application is still distant. Secondly, there are numerous molecular mechanisms downstream of KIAA1429 that remain unexplored; the ceRNA mechanism of EBLN3P in radioresistance is also insufficiently investigated. Finally, whether other target genes downstream of miR-153-3p exist is still unknown and requires further exploration and validation. In the future, verifying the above functional mechanisms in animal models and investigating the downstream mechanisms of miR-153-3p are critical areas for further research to improve the management of CRC.

## Data Availability

The datasets supporting the conclusions of this article are presented in the article. Further inquiries can be directed to the corresponding author.
